# Orthodontic Treatment in Idiopathic Root Resorption: A Narrative Review and a Clinical Case Report

**DOI:** 10.3390/jcm15114074

**Published:** 2026-05-25

**Authors:** Marta Karolczuk, Ilona Radej, Irena Grodzka, Antonino Lo Giudice, Izabela Szarmach

**Affiliations:** 1 Department of Orthodontics, Medical University of Białystok, ul.Waszyngtona 15A, 15-274 Białystok, Polandirena.grodzka@umb.edu.pl (I.G.); izabela.szarmach@umb.edu.pl (I.S.); 2 Section of Pediatric Dentistry, Department of Medical-Surgical Specialties, School of Dentistry, University of Catania, Via Santa Sofia 78, 95123 Catania, Italy; antonino.logiudice@unict.it

**Keywords:** root resorption, orthodontics, interceptive, malocclusion, Angle Class III, orthodontic anchorage procedures, extraoral traction appliances, tooth, supernumerary

## Abstract

Idiopathic root resorption is diagnosed when external root resorption occurs in the absence of an identifiable etiological factor. Two main forms are described in the literature: apical and cervical. Owing to the rarity of this condition and the limited number of published reports, evidence-based recommendations for orthodontic management are currently lacking. The aim of this study was to provide a narrative overview of published case reports describing orthodontic procedures performed in patients with idiopathic root resorption and to supplement the available literature with a detailed clinical case. A case of a 7-year-7-month-old female patient presenting with generalized idiopathic root resorption and a concomitant skeletal Class III malocclusion is described. In this patient, skeletal anchorage was used to support maxillary protraction in an attempt to obtain an orthopedic effect. The literature review had a narrative character and was based on a structured search of the PubMed, Scopus, and Web of Science databases covering the period from January 2010 to December 2025. Only English-language case reports meeting strict eligibility criteria were considered. Of 47 records initially identified, two fulfilled the inclusion criteria; an additional two case reports were retrieved through manual searching. Conclusions: Given that the available evidence is limited to isolated case reports and a single clinical observation, the present findings do not allow for reliable conclusions regarding the safety, effectiveness, or general applicability of orthodontic treatment in patients with idiopathic root resorption. Clinical management should therefore be individualized, with careful documentation and close radiological follow-up. Further well-documented clinical reports are required to better characterize treatment-related risks in this patient group.

## 1. Introduction

Root resorption is a physiological or pathological process involving progressive loss of dentin and root cementum due to the activity of resorptive cells—dentinoclasts and cementoclasts [1,2]. When excessive osteoclastic activity exceeds the reparative capacity of osteoblasts, resorption ensues [1]. Physiologically, it occurs in the primary dentition and enables tooth exfoliation, whereas in the permanent dentition, it is most often pathological in nature [1,3].

The triggers of resorption can be classified as systemic and local. Systemic factors include: hyperparathyroidism, hypoparathyroidism, hyperphosphatemia, hypophosphatemia, Gaucher disease, Paget’s disease of bone, Goltz syndrome, Papillon–Lefèvre syndrome, anachoresis, Turner syndrome, as well as dietary habits associated with endocrine and systemic disorders [4]. Local factors include: orthodontic treatment, trauma, periapical or periodontal inflammation, tumors, cysts, occlusal overload, impacted and supernumerary teeth, transplantation, and tooth replantation [4,5]. Additional causes may include infections, localized pressure, high temperature, and certain esthetic dental procedures [1,6]. 

If none of the above factors meet the criteria and no cause can be identified, the condition is termed idiopathic tooth resorption [1,3,5,6,7,8]. 

Idiopathic resorption may involve either the apical or cervical region of the tooth. Cervical resorption begins near the cementoenamel junction and progresses toward the pulp. In the apical form, resorption starts at the apex and advances coronally, resulting in gradual shortening and rounding of the remaining root [5,7,9]. 

It may affect a single tooth or—less commonly—the entire dentition [10]. The literature emphasizes that idiopathic, multifocal resorption (MIR) represents a particularly aggressive pattern that often remains undetected on clinical examination and, in most cases, ultimately leads to tooth extraction [11]. Despite detailed clinical and radiological descriptions, the etiology and natural course of idiopathic tooth resorption remain incompletely understood.

Idiopathic tooth resorption can be associated with other dental anomalies, such as early loss of primary teeth, agenesis, dens invaginatus, conical teeth, supernumerary teeth, microdontia, taurodontism, and pulp chamber calcifications [12]. 

Patients are usually diagnosed during routine panoramic radiography, which reveals features of external resorption and is essential for evaluation. A thorough medical and dental history typically fails to disclose an identifiable cause. Patients are often asymptomatic, although tooth mobility may occasionally be observed. A positive pulp vitality test is usually recorded for the affected teeth [13]. Although the distribution of lesions varies across reports, teeth involved in this pathology often share similar phenotypic characteristics: short premolar roots; short, tapered incisor roots; and short distal roots of the mandibular first molar [14]. 

Orthodontic treatment is considered a potential accelerator of the resorptive process; however, clear management guidelines for such cases are lacking [14]. Available evidence regarding orthodontic intervention in patients with idiopathic root resorption is limited and largely restricted to isolated case reports. Importantly, it remains unclear to what extent modifications of orthodontic biomechanics—such as force magnitude, anchorage selection, treatment duration, and monitoring protocols—may influence the risk of further resorption during treatment. Several authors suggest that applying light forces and shortening the duration of active treatment—by using appropriate techniques—may favorably influence the prognosis of teeth following therapy [15]. 

In view of the rarity of idiopathic root resorption and the absence of established guidelines for orthodontic management, the inclusion of a clinical case report represents a necessary complement, illustrating the practical application of the limited evidence currently available.

Given the limited and predominantly case-based nature of the available literature, this paper aims to provide a focused overview of published data addressing orthodontic treatment in patients with idiopathic root resorption and to supplement this overview with the presentation of an original clinical case of a female patient with generalized idiopathic tooth resorption and a concomitant skeletal Class III malocclusion.

## 2. Data Description

### 2.1. Study Design

This study combines a focused narrative review of the published case-based literature on orthodontic treatment in patients with idiopathic root resorption with a clinical case report illustrating diagnostic and therapeutic challenges in a patient with generalized idiopathic root resorption and a Class III malocclusion.

### 2.2. Review Guidelines

This study was conducted as a narrative review. The review process was designed to provide a focused, interpretative overview of selected case-based publications rather than an exhaustive or protocol-driven synthesis. The protocol was not registered in a public registry.

### 2.3. Selection Criteria

Inclusion criteria:

− Articles published in English;
− Publications from 2010 to 2025;
− Case reports and case series of idiopathic root resorption in which orthodontic treatment was performed.

Exclusion criteria:

− Resorption caused by orthodontic or endodontic treatment;
− Resorption occurring in the course of systemic diseases.

### 2.4. Eligibility Criteria

The review was conducted as a narrative appraisal of case-based publications describing orthodontic treatment in patients with idiopathic root resorption. Studies were selected based on clinical relevance to the research objective, without applying a formal eligibility framework.

### 2.5. Search Strategy

The review was informed by a focused and selective literature search conducted in three databases: Web of Science, Scopus, and PubMed. The search covered publications from January 2010 to 29 December 2025 and was restricted to articles available in English. The search strategy was intentionally narrow and designed to identify clinically relevant case-based reports, rather than to comprehensively capture all available literature on idiopathic root resorption. Accordingly, the applied search terms should be interpreted as supporting a targeted selection of representative reports rather than as a sensitive or exhaustive retrieval strategy. The following search phrase was used: (“idiopathic root resorption” OR “generalized root resorption”) AND orthodontic.

The literature search and relevance-based selection of publications were performed independently by two authors. Any discrepancies regarding eligibility were resolved through discussion and consensus.

### 2.6. Selection of Articles and Data Collection

**Figure 1:**
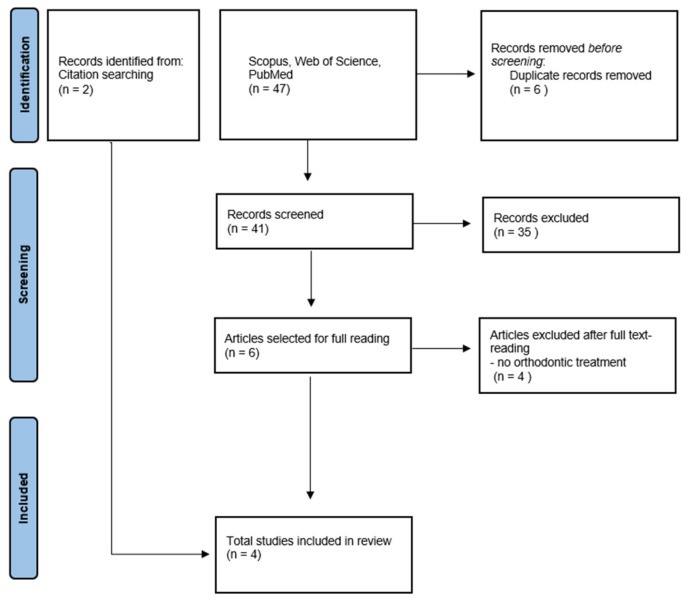
PRISMA flow diagram.

Titles and abstracts of potentially relevant records were first screened. Articles that satisfied the eligibility criteria then underwent full-text assessment. The study selection is depicted in the PRISMA flow diagram (Figure 1). Besides database searches, hand-searching was conducted to capture any additional relevant publications.

### 2.7. Clinical Case

Clinical data were collected from a young female patient treated at the Department of Orthodontics of a university-based dental hospital between June 2022 and March 2026. The dataset comprised intraoral and extraoral photographs, digital dental models, panoramic radiographs, and cephalometric analyses; cone-beam computed tomography (CBCT) was not performed.

A detailed medical history was obtained from the patient’s mother, and available medical records were reviewed. In addition, a pediatric consultation was conducted to exclude systemic conditions and other potential etiological factors associated with root resorption. Based on the medical history and clinical assessment, no evidence of systemic disease, trauma, or previous orthodontic treatment was identified. Written informed consent for the use of clinical and radiographic data for scientific purposes and publication was obtained from both the patient and her parent.

## 3. Results

### 3.1. Selection of Articles

The search identified 47 records (Web of Science: 33; Scopus: 7; PubMed: 7). After deduplication (n = 6), 41 unique records remained for screening. Screening was performed based on titles and abstracts. Six articles were eligible for full-text assessment. Two of the full-text articles met the inclusion criteria (Table 1), as they reported orthodontic treatment in patients with idiopathic root resorption, while 39 were excluded due to the absence of orthodontic treatment in the reported cases or a different etiology (most commonly orthodontic- or endodontic-related). In addition, two studies identified through manual searching were included, as they reported orthodontic management in patients with idiopathic root resorption. The selection process is presented in the PRISMA flow diagram (Figure 1), and detailed synthesis of key elements is provided in Table 2.

#### 3.1.1. Quality Assessment

The methodological quality of the included case reports was appraised using the Joanna Briggs Institute (JBI) Critical Appraisal Checklist for Case Reports. The checklist consists of eight items addressing key aspects such as patient characteristics, clinical history, diagnostic assessment, therapeutic intervention, reporting of adverse events, and follow-up. Each item was assessed descriptively using the categories “yes,” “no,” “unclear,” or “not applicable.” The purpose of this appraisal was not to exclude studies, but to contextualize the strengths and limitations of the available case-based evidence. Detailed appraisal results are provided in the Supplementary Materials (Tables S1–S4).

#### 3.1.2. Narrative Synthesis of Included Case Reports

A total of four case reports describing orthodontic treatment in patients diagnosed with idiopathic root resorption were identified and narratively synthesized. The mean patient age was 15.25 years (range: 14–17), and all reported cases involved female patients. The most frequently described malocclusion types were Angle Class I and Class II (two cases each). Clinical presentation varied substantially, ranging from asymptomatic findings to incisor mobility, pain, or the presence of a “pink spot” in affected teeth. Diagnostic imaging modalities differed across reports and included cone-beam computed tomography (CBCT), panoramic radiographs (OPG), cephalometric analyses, and intraoral or periapical radiographs, with variable sensitivity for detecting subtle longitudinal root changes.

Three broad management approaches were described: orthodontic treatment alone using fixed appliances and light forces (two cases), combined orthodontic–surgical management using a surgery-first protocol (one case), and multidisciplinary care integrating prosthetic, surgical, and orthodontic interventions (one case). Across the included reports, no radiographic evidence of progression of root resorption was described on follow-up imaging during the reported treatment periods. Improvements in occlusal relationships and smile esthetics were reported by the original authors in all cases.

Authors consistently highlighted the use of cautious biomechanical strategies, including low force application, staged activation, and avoidance of excessive or unnecessary loading. These approaches were described in the included reports; however, reporting heterogeneity, differences in imaging modalities, and the descriptive nature of the data precluded any inference regarding their effect on the course of root resorption. Due to inconsistent documentation, it was not possible to standardize the number or distribution of teeth affected across reports. The overall level of evidence was limited to individual case reports without control groups or standardized outcome measures, restricting generalizability.

Taken together, this narrative synthesis indicates that individualized orthodontic approaches have been described in the literature as being applied in patients with idiopathic root resorption, with radiographic monitoring forming a common component of clinical follow-up. These observations reflect reported case-level experiences based on descriptive and observational reporting rather than uniform treatment recommendations or standardized outcome assessments.

### 3.2. Clinical Case

A 7-year-7-month-old female patient was referred by another orthodontist to the orthodontic clinic due to generalized tooth resorption. The principal findings were marked mobility of tooth 22, a reverse overjet in the incisor region, and premature eruption of permanent teeth. The medical/dental history obtained from the patient’s mother did not reveal any factors that could account for the resorption; the child had no known systemic diseases and was not taking any medications or dietary supplements.

**Figure 2:**
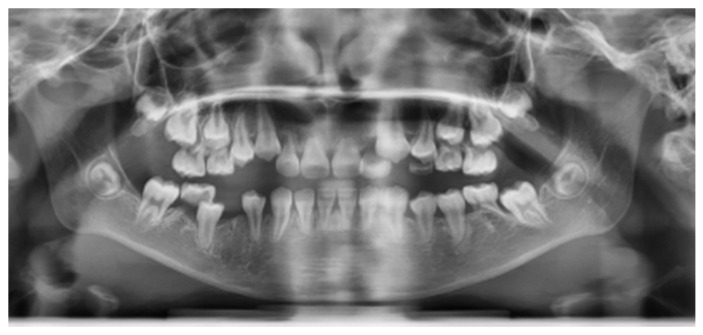
OPG at age 7 years 3 months.

On a panoramic radiograph taken 4 months before the initial visit (Figure 2), teeth 16 and 26 exhibited advanced resorption involving the entire root length. Cervical radiolucencies were noted in teeth 46 and 36, with rarefaction and blurred root contours, more pronounced at 46. Generalized apical shortening was visible, with particularly advanced changes in teeth: 12, 11, 21, 22, 32, 31, 41, and 42.

Extraoral examination showed a symmetrical face, concave profile, and a negative lip step (Korkhaus). Intraoral examination revealed pathologic mobility of 16, 26, and 22, physiologic mobility of 55 and 65, and absence of 46. Clinically, a reverse overjet (anterior crossbite), reduced overbite, and posterior open bite were recorded.

A detailed general medical history revealed neonatal jaundice secondary to subcutaneous hemorrhages (ICD-10: P58.0) and a congenital atrial septal defect (ICD-10: Q21.1). The patient was otherwise healthy, was not taking medications or dietary supplements, and had been vaccinated according to the national immunization schedule. A history of SARS-CoV-2 infection was noted; follow-up vitamin D levels during this period remained within normal limits.

An extensive diagnostic workup was undertaken to exclude systemic, metabolic, infectious, and other potential causes of root resorption. A comprehensive laboratory panel performed at the age of 4 included inflammatory markers, coagulation parameters, cardiac biomarkers, a metabolic and mineral profile, thyroid function tests, serum vitamin D levels, and complete blood count; all results were within normal reference ranges. Additional testing for Lyme disease and parasitic infections yielded negative results.

Further diagnostic evaluation included imaging and functional studies. Brain magnetic resonance imaging (MRI), electroencephalography (EEG), and chest radiography revealed no abnormalities. Review of the available medical records and pediatric consultation did not identify any underlying systemic condition that could account for the observed dental findings. The patient was also referred to a genetic clinic; however, genetic testing was not performed, as no specific candidate gene or clearly defined indication for targeted molecular analysis could be established.

Based on the absence of identifiable local, systemic, or clearly defined genetic etiological factors, a working diagnosis of idiopathic root resorption was established.

**Figure 3:**
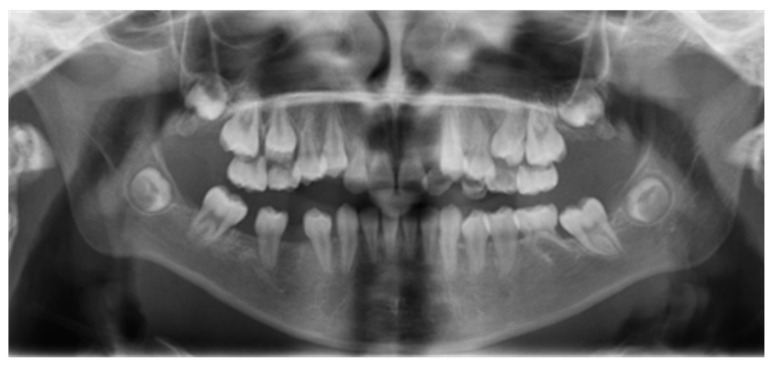
OPG at age 7 years 7 months.

Given the degree of tooth mobility and the absence of tooth 46, a follow-up panoramic radiograph was obtained four months after the baseline image to document the current dental status (Figure 3). This control radiograph demonstrated residual roots of tooth 46 and radiographic features consistent with resorptive involvement of teeth 16, 26, and 22.

**Figure 4:**
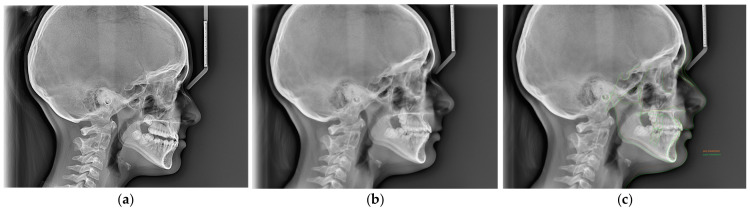
(**a**) Pre-treatment lateral cephalogram; (**b**) post-treatment lateral cephalogram; (**c**) post-treatment superimposition. Pre- and post-treatment cephalometric tracings were performed by the same examiner using NemoCeph software, 2022 version (Nemotec, Madrid, Spain). For verification purposes, the results were compared with an independently performed cephalometric analysis using a different software platform, showing no clinically relevant discrepancies.

**Figure 5:**
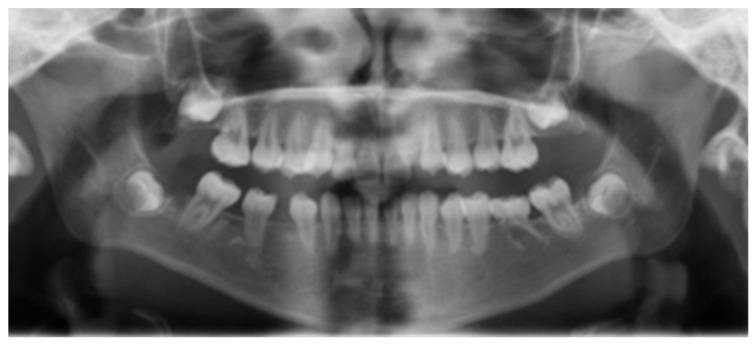
OPG at age 8 years 3 months.

**Figure 6:**
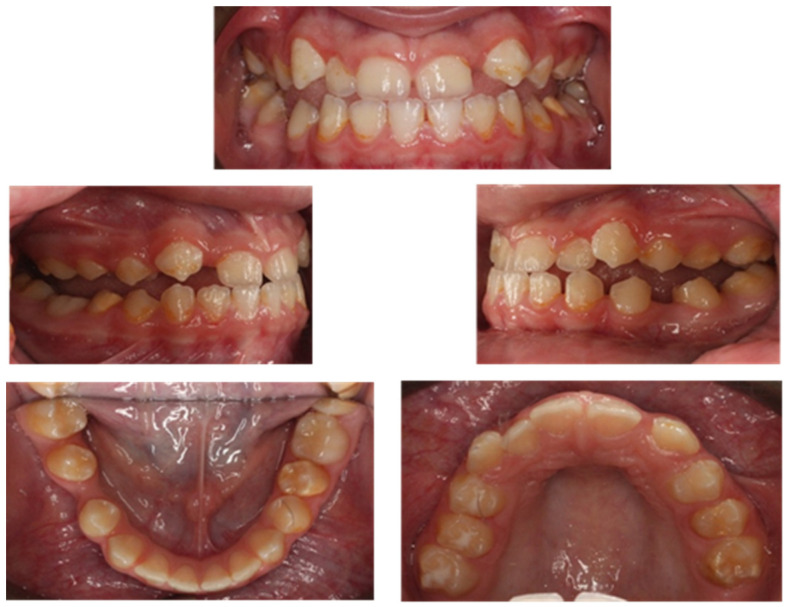
Intraoral photographs before treatment at age 8 years 3 months.

**Figure 7:**
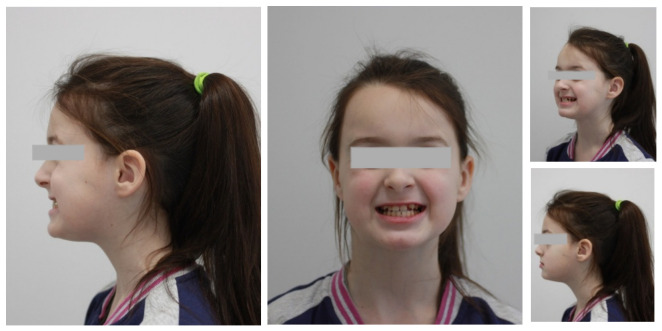
Extraoral photographs before treatment at age 8 years 3 months.

**Figure 8:**
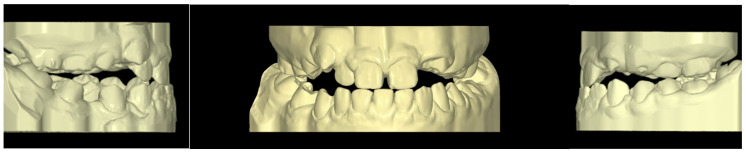
Digital diagnostic models before treatment at age 8 years 3 months.

After six months of observation, the patient lost teeth 16, 22, and 26 due to advanced resorption. Histopathologic examination was not performed, as no dental specimens were available for pathological assessment. The remaining permanent teeth demonstrated physiologic mobility. The patient was scheduled for further diagnostic assessment. Referrals were issued for a lateral cephalogram (Figure 4) and a new panoramic radiograph (Figure 5); clinical photographs were taken (Figure 6 and Figure 7) and impressions were obtained for diagnostic models (Figure 8).

On the comparative panoramic radiograph obtained at six months, germ formation of teeth 18, 28, 38, and 48 was evident. Tooth 36 exhibited combined apical and cervical radiographic features of resorption with infra-position relative to adjacent teeth, suggestive of early re-inclusion. Radiographic features of apical resorption were documented on panoramic imaging in the remaining teeth.

Cephalometric analysis confirmed skeletal Class III per WITS, attributed to maxillary retrognathia. Vertical parameters were within normal limits. A slight proclination of maxillary incisors and an anterior position/proclination of mandibular incisors relative to the APg line were observed (Table 3).

Considering the traumatic anterior occlusion and following radiological review, model analysis, and facial assessment, interceptive facemask therapy was initiated. Owing to the presence of generalized root resorption and an underlying skeletal Class III pattern, a facemask anchored to surgically placed miniplates in the oral vestibule was selected to provide skeletal anchorage.

Titanium miniplates were contoured intraoperatively to adapt to the local anatomy and fixed on both sides of the apertura piriformis and on the lateral nasal wall of the maxilla. The hook-shaped extensions were positioned to emerge into the oral vestibule, allowing attachment of the extraoral traction system. The facemask elastics were applied at an angle of approximately 30° relative to the occlusal plane.

Facemask therapy was delivered for approximately 12 months. Radiographic monitoring of root resorption was performed using serial panoramic radiographs, including a control examination at 8 months after initiation of facemask therapy and a subsequent follow-up at 2.5 years from the start of treatment. No formal scoring system was applied; therefore, the assessment was observational.

Patient compliance was assessed clinically and was considered very good, with the patient adhering to the recommended facemask wear time of at least 12 h per day, as confirmed during regular follow-up visits.

**Figure 9:**
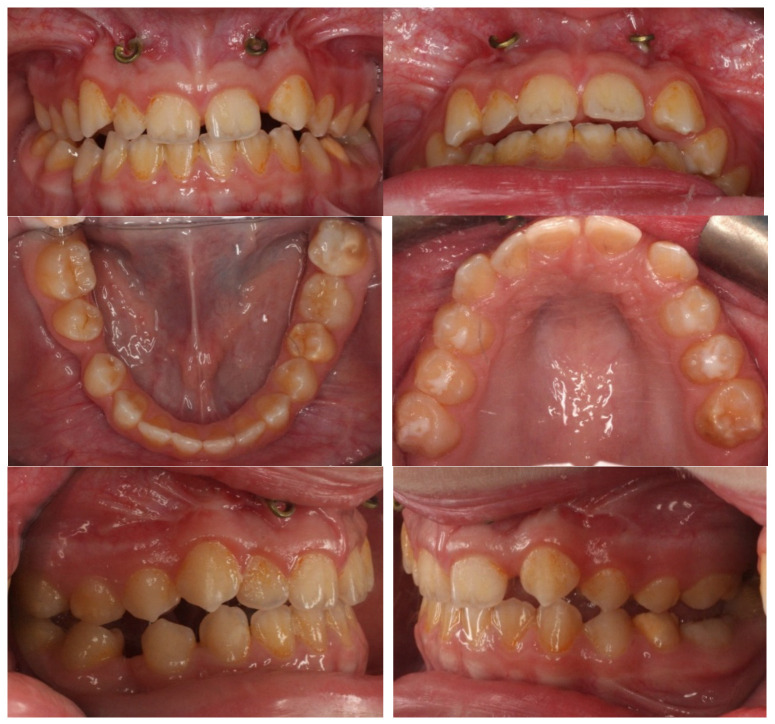
Intraoral photographs after 8 months of facemask therapy at age 9 years.

**Figure 10:**
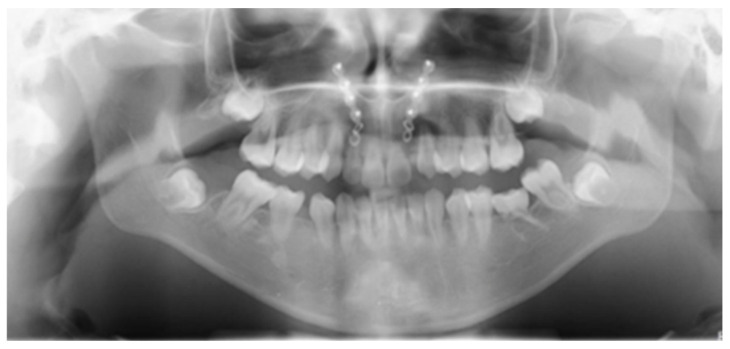
OPG after 8 months of facemask therapy at age 9 years.

**Figure 11:**
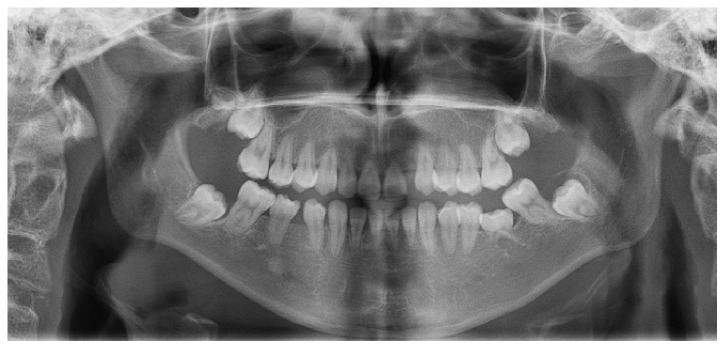
OPG after 2 years 6 months of facemask therapy at age 11 years.

**Figure 12:**
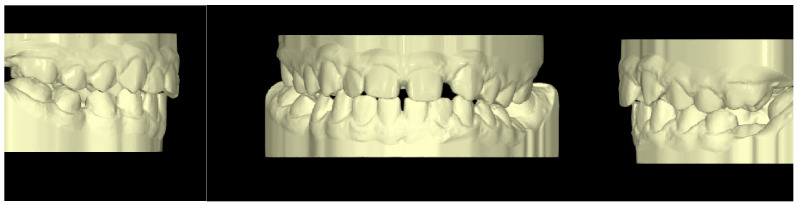
Digital diagnostic models after completion treatment at age 11 years.

**Figure 13:**
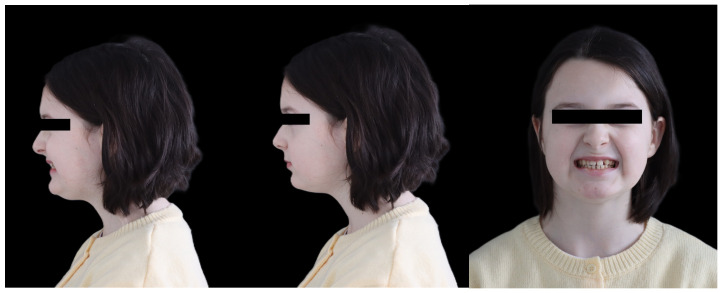
Extraoral photographs after treatment at age 11 years.

Force magnitude was controlled at 400–500 g, increased stepwise (initially ½″ 14 oz, then 5/16″ 14 oz elastics), with instructions to wear elastics 12 h/day. At scheduled follow-ups, dental status was monitored and overjet was recorded. After 2 months, a positive overjet of 0.5 mm and premolar contacts were established; progressive re-inclusion of 36 was noted. At 6 months, molar contacts were present with a pronounced anterior open bite; facemask use was continued. After 8 months of maxillary protraction, overjet reached 2.0 mm (Figure 9). On a control panoramic radiograph obtained during treatment (Figure 10), re-inclusion of tooth 36 was visible; within the limits of panoramic imaging and based on descriptive assessment, no radiographic signs of progression of apical root resorption were observed in the remaining teeth. At 12 months, facemask therapy was completed, yielding a positive overjet of 3.5 mm. New impressions and photographs were obtained. At 2.5 years from the start of protraction, another control panoramic (Figure 11) and cephalometric radiograph (Figure 4) were taken, together with updated photographs and scan (Figure 12 and Figure 13). The panoramic radiograph demonstrated a developing supernumerary tooth 19; no visually apparent features suggestive of progression of root resorption were identified on panoramic imaging.

Overall, treatment resulted in a clinically meaningful anteroposterior correction—from a reverse overjet at baseline to a positive overjet of 3.5 mm—with observable improvements in facial profile and occlusion. Within the limitations of panoramic radiographic imaging and based on descriptive assessment, serial radiographs did not demonstrate evidence of progression of root resorption during therapy. Radiographic findings were documented over time, with notable changes limited to progressive infraocclusion of tooth 36 and the emergence of a supernumerary tooth 19. Post-treatment cephalometrics demonstrated improvements in WITS and SNA (Table 4, Figure 4). The treatment summary is presented in Table 5.

## 4. Discussion

Idiopathic root resorption is an uncommon condition, and the available literature is largely limited to individual case reports and small case series. Consequently, standardized, evidence-based management guidelines are lacking, which makes the development of uniform treatment protocols challenging [12,15]. Most published data are descriptive in nature, precluding definitive conclusions or generalized clinical recommendations.

It should be noted that some cases previously classified as idiopathic may later be attributed to specific etiologic factors, such as viral infections affecting peripheral nerves [14]. Similarly, cases demonstrating a familial pattern—although currently considered idiopathic—may ultimately be reclassified as genetically determined once their molecular background is clarified [11]. These considerations may partly explain the limited number of eligible studies identified in the present review.

From the perspective of potential predisposing factors, Wang et al. suggested that alterations in female hormonal balance might contribute to the development and progression of multiple idiopathic cervical root resorption (MICRR) [18]. Such observations are biologically plausible and appear to be consistent with features reported in some of the included cases, including a predilection for younger individuals and preferential involvement of premolars at early stages. A characteristic radiographic finding described in MICRR is the “apple-core”-type lesion at the cementoenamel junction. In terms of imaging, cone-beam computed tomography (CBCT) has been advocated as a useful adjunct for diagnosis and assessment of lesion extent, thereby supporting individualized treatment planning [18]. However, in pediatric patients, CBCT use requires careful justification due to radiation considerations.

The findings of the literature review did not allow the identification of a single, clearly defined diagnostic or therapeutic pathway, given the limited and predominantly case-based nature of the available evidence. Quality appraisal using the JBI checklist further contextualized these findings by revealing several recurrent methodological limitations across the included case reports. Most notably, heterogeneity and incompleteness in reporting diagnostic criteria for idiopathic root resorption, limited detail regarding orthodontic treatment protocols, insufficient documentation of adverse events, and variable or absent follow-up were observed. These factors substantially constrain the interpretability and generalizability of the published data and reinforce the need for cautious clinical extrapolation.

In the present case, the patient was in a growth phase and at the mixed-dentition stage, resulting in a dynamic clinical situation that required close monitoring. Richert et al. [11] reported that aggressive forms of idiopathic resorption—particularly cervical variants—are frequently associated with tooth loss, often culminating in extraction. In this case, loss of the first permanent molars was observed and was most likely related to extensive resorptive changes; however, a causal relationship cannot be established with certainty. Notably, the resorptive process demonstrated a heterogeneous pattern, with selective progression to severe destruction and tooth loss involving the previously mentioned teeth, as well as loss of tooth 22, for which it was not possible to reliably determine the resorption type. At the same time, the remaining teeth predominantly showed apical shortening, without clinically evident complications during the observation period. This observation suggests a non-uniform expression of the disease across the dentition, an aspect that should be taken into account when interpreting clinical progression in idiopathic root resorption. It should be emphasized that radiographic follow-up in the present case was based on descriptive, observational assessment of serial panoramic radiographs. Given the limited sensitivity of panoramic imaging for detecting subtle root changes, the absence of radiographic evidence of progression should not be interpreted as evidence of biological stability of the resorptive process.

An additional complicating factor was the presence of a skeletal Class III malocclusion, which represents a well-recognized therapeutic challenge in pediatric and adolescent patients [19]. After comprehensive diagnostic evaluation confirming maxillary retrognathia, maxillary protraction was selected as a reasonable orthopedic option in this case.

Conventional facemask therapy has known limitations, including dentoalveolar side effects such as incisor proclination, molar extrusion and mesial tipping, and an increased risk of canine impaction [20]. In this clinical context, a hybrid rapid palatal expander combined with a facemask was not selected, as even appliances incorporating skeletal anchorage may still transmit a portion of the applied forces to the dentition. As demonstrated by Maino et al. [21], the use of two mini-implants was still associated with mesialization of the maxillary first molars, albeit of limited magnitude, underscoring the presence of residual dental effects with such protocols.

Given the patient’s generalized root resorption, reduction of additional tooth-borne loading was considered a precautionary element of the clinical reasoning in this case. Based on this clinical reasoning, a facemask connected to surgically placed miniplates was selected as the anchorage approach for this patient. A comparable miniplate-assisted maxillary protraction protocol has been described by Liang et al. [22]. Palatal skeletal anchorage has also been proposed as an alternative approach and, according to Raghib et al. [20], may contribute to shorter treatment duration. While these approaches are biomechanically rational and aim to reduce dental loading, the evidence supporting their use in patients with idiopathic root resorption remains limited to isolated reports.

Based on reported case-level experiences, orthodontic intervention has been discussed as a possible option in carefully selected cases to address functional or esthetic concerns; however, such decisions must be balanced against the potential risk of further resorption and the need for cautious biomechanical planning.

Within the available case-based literature, orthodontic forces have been suggested as a potential factor that may influence the progression of root resorption [12,15]. Accordingly, strategies such as the application of relatively light forces and reduction of active treatment time have been described in the literature, primarily based on expert opinion and anecdotal evidence. Adjunctive surgical approaches, including corticotomy or osteotomy, have been described as means to accelerate treatment via the regional acceleratory phenomenon (RAP). In patients with completed growth and significant dentofacial deformities, a Surgery First approach has also been discussed [15]. However, none of these approaches can currently be regarded as evidence-based recommendations for patients with idiopathic root resorption. Careful patient selection, detailed diagnostic work-up, cautious force application, and thorough informed consent remain essential components of clinical decision-making.

The limitations of this review must be emphasized. The primary constraint is the very small number of available publications addressing idiopathic root resorption in the context of orthodontic treatment, reflecting the rarity of the condition. Additional limitations include restriction to English-language publications, a narrow search strategy, absence of standardized outcome measures, and inherent diagnostic uncertainty associated with the “idiopathic” classification, all of which may influence the assembled evidence base. Because only case reports were eligible for inclusion, the findings should be interpreted with considerable caution.

Further publication of well-documented clinical cases—with transparent diagnostic criteria, standardized radiographic assessment, and long-term follow-up—may help improve collective understanding of this condition and support more informed clinical reasoning in the future.

## 5. Conclusions

Idiopathic root resorption is a rare condition that poses substantial diagnostic and therapeutic challenges, and standardized management guidelines are lacking. Based on the limited, case-based evidence available, the included reports allow us to hypothesize that, under carefully selected conditions, orthodontic treatment may allow partial esthetic and functional objectives to be achieved without radiographic features suggestive of progression of pre-existing root resorption.

The present case illustrates that interceptive treatment using a facemask supported by skeletal anchorage may be considered as a potential treatment approach for correcting Class III malocclusion in selected patients at increased risk of resorption, while emphasizing that conclusions regarding safety and effectiveness cannot be generalized.

An important clinical consideration in reported cases is the minimization of tooth-borne loading, for example, through the use of skeletal anchorage and careful staging of mechanics with close radiographic monitoring throughout treatment. Interdisciplinary collaboration may be beneficial in complex cases, including the selective use of adjunctive surgical procedures such as osteotomy or corticotomy; however, such approaches should be regarded as case-dependent rather than standard recommendations.

## Figures and Tables

**Table 1:** Summary of included articles.

No.	Identifier	Title	Authors	Journal/Year	Database
1	DOI:10.1590/2176-9451.20.1.108-117.oar	Orthodontic treatment in patient with idiopathic root resorption	Rey D, Martínez Smit R, Gamboa L [12]	Dental Press J Orthod, 2015	Scopus
2	DOI:10.1016/j.jormas.2018.11.005	Orthodontic–Surgical management in a class II case with idiopathic root resorption	Adélaïde C, de C. Elke V, Ria V, Nasser N [15]	J Stomatol Oral Maxillofac Surg, 2019	Scopus
3	DOI: 10.1111/scd.12539	Multiple idiopathic cervical root resorption: Case report of an unusual presentation	Samara, E. Kelly, E. Walker, R. Borumandi, F. [16]	Special Care in Dentistry, 2021	Scopus
4	10.5152/TurkJOrthod.2016.15-00023R1	The Orthodontic Treatment of a Patient with Idiopathic Root Resorption in Upper Incisors	Çelik, Ö Ersöz, M Kamalak, H [17]	Turkish Journal of Orthodontics, 2015	Web of Science

**Table 2:** Descriptive summary of included case reports.

Authors	Year	Age/Sex	Malocclusion Type	Clinical Signs	Imaging	Circumstances of Diagnosis	Teeth Involved	Orthodontic/Adjunctive Management	Outcome
Rey, D.; Martínez Smit, R.; Gamboa, L. [12]	2015	17 y, female	Class I; protrusion of maxillary and mandibular incisors; spacing/diastema	Pain in posterior teeth during mastication; mobility of tooth 22	Cephalometric analysis; panoramic radiograph; periapical radiographs)	Referral from another orthodontist	Generalized involvement; pronounced resorption at 12, 22, 44, 45	Fixed appliance with light forces; incisors joined later in treatment	Esthetic improvement was observed during treatment, with no radiographic signs suggestive of progression of root resorption on serial panoramic radiographs.
Adélaïde, C.; Engels, V. de C.; Vervoort, R.E.; Nasser, N. [15]	2018	14 y, female	Class II/2; retrognathic mandible; OJ = 2 mm; OB = 4 mm; anterior crowding	Mobility of tooth 22	CBCT, cephalometric analysis	Identified during CBCT assessment	Marked resorption of premolars and incisors	Orthodontic–surgical approach using a surgery-first protocol with osteotomy support	Clinically relevant therapeutic improvement was achieved, with no radiographic signs suggestive of progression of root resorption on post-treatment panoramic radiographs.
Samara, E.; Kelly, E.; Walker, R.; Borumandi, F. [16]	2020	16 y, female	Class II/2; maxillary anterior crowding	Dull, continuous pain; slight mobility of lower incisors; trauma to lower incisors 7 months prior; pink spot on 43 and 44; positive ethyl-chloride test; mobility grade II (32, 33, 44); normal percussion sound; BOP+ at mobile teeth	panoramic radiograph; CBCT	Referred by a general dental practitioner	Pronounced cervical resorption in 12 teeth (mandibular arch) and 3 teeth in the first quadrant (14, 15, 16)	Multidisciplinary management (prosthetic, surgical and orthodontic)	On a panoramic radiograph obtained 4.5 years post-treatment, normal implant function was observed, with no radiographic evidence of progression of root resorption in teeth 37 and 16. The patient is awaiting final prosthetic restorations.
Çelik, Ö.; Ersöz, M.; Kamalak, H. [17]	2015	14 y, female	Class I (Angle); bilateral canine Class II; skeletal Class III; crossbite on 11; crowding	No complaints; occlusal trauma on tooth 11	OPG; intraoral radiographs; cephalometric analysis	Incidental finding on routine panoramic radiograph	Apical resorption of maxillary incisors	Upper-arch fixed appliance: 0.012″ round Ni-Ti → 0.016″ Ni-Ti → 0.018″ Ni-Ti; lower arch not treated to shorten active time; lower discluding splint used	Improvement in esthetic outcome was observed, with no radiographic evidence of progression of root resorption on follow-up panoramic radiographs.

**Table 3:** Pre-treatment cephalometric measurements.

Parameter	Norm (±SD)	Patient Value
SNA (°)	82.0 ± 3.5	77.0
SNB (°)	79.0 ± 3.0	77.0
ANB (°)	3.0 ± 2.5	0
Wits (mm)	0.0 ± 2.0	−3.8
ML-NL (°)	25.0 ± 6.0	28.0
ML–NSL (°)	33.0 ± 6.0	34.0
NL–NSL (°)	8.0 ± 3.0	7.0
1+: NL (°)	110.0 ± 6.0	114.0
1−: ML (°)	94.0 ± 7.0	91.0
1−: APg (mm)	1.0 ± 2.0	3.8

**Table 4:** Post-treatment cephalometric measurements.

Parameter	Norm (±SD)	Patient Value
SNA (°)	82.0 ± 3.5	80.0
SNB (°)	79.0 ± 3.0	77.0
ANB (°)	3.0 ± 2.5	3.0
Wits (mm)	0.0 ± 2.0	3.2
ML-NL (°)	25.0 ± 6.0	30.0
ML–NSL (°)	33.0 ± 6.0	35.0
NL–NSL (°)	8.0 ± 3.0	5.0
1+: NL (°)	110.0 ± 6.0	116.0
1−: ML (°)	94.0 ± 7.0	92.0
1−: APg (mm)	1.0 ± 2.0	0.4

**Table 5:** Case summary.

Parameter	Information
Age at presentation	7 years 7 months
Main findings	Mobility of tooth 22; traumatic anterior occlusion on the incisors
History	Premature eruption of permanent teeth; no systemic diseases or trauma
Diagnosis	Generalized idiopathic root resorption; skeletal Class III malocclusion
Pre-treatment tooth loss	Missing tooth 46 at presentation, loss of teeth 16, 22, 26 due to advanced resorption during diagnostic evaluation
Imaging	Panoramic radiographs (Figure 2, Figure 3, Figure 5, Figure 10 and Figure 11); cephalometry (Figure 4); clinical photographs (Figure 6 and Figure 7)
Pre-treatment cephalometric findings	Skeletal Class III; maxillary retrognathia SNA= 77°; negative WITS= −3.8 mm
Treatment	Facemask therapy supported by surgically placed miniplates (skeletal anchorage)
Duration of facemask therapy	12 months
Outcomes of facemask therapy	Overjet: initially negative → 0.5 mm (after 2 months) → 3.5 mm (after 12 months). No tooth loss; radiographic appearance of pre-existing root resorption did not show clearly evident changes during facemask therapy
Post-treatment cephalometric findings	Improvements in WITS = 3.2 mm and SNA= 80°

## Data Availability

No new data were created or analyzed in this study. Data sharing is not applicable to this article.

## Supplementary Materials

The following supporting information can be downloaded at: https://www.mdpi.com/article/10.3390/jcm15114074/s1, Table S1. JBI Critical Appraisal Checklist for Case Reports—Article 1; Table S2. JBI Critical Appraisal Checklist for Case Reports—Article 2; Table S3 JBI Critical Appraisal Checklist for Case Reports—Article 3; Table S4. JBI Critical Appraisal Checklist for Case Reports—Article 4.
