# Supplementary material for: Orthodontic Treatment in Idiopathic Root Resorption: A Narrative Review and a Clinical Case Report
Source: J Clin Med. 2026 May 25;15(11):4074. doi: 10.3390/jcm15114074 (PMC13257550; doi:10.3390/jcm15114074)
Supplement: Supplementary file 1 [file jcm-15-04074-s001.zip › jcm-4250761-supplementary.pdf]

JBI Critical Appraisal Checklist for Case Reports

Article 1

Rey D, Martínez Smit R, Gamboa L. Orthodontic treatment in patient with idiopathic root resorption.

DOI: 10.1590/2176-9451.20.1.108-117.oar

Table S1. JBI Critical Appraisal Checklist for Case Reports – Article 1

No.	JBI Critical Appraisal Question	Response	Explanation
1.	Were the patient's demographic characteristics clearly described?	**Yes**	The patient's age (17 years) and sex (female) were clearly reported.
2.	Was the patient's history clearly described and presented as a timeline?	**Yes**	The clinical history and treatment sequence were described in a logical order, allowing reconstruction of the course of events.
3.	Was the current clinical condition of the patient on presentation clearly described?	**Yes**	The initial clinical condition, including evidence of root resorption, was clearly outlined at presentation.
4.	Were diagnostic tests or assessment methods and the results clearly described?	**Unclear**	Although radiographic findings illustrating root resorption were presented, the diagnostic criteria and the systematic exclusion of secondary causes were not fully detailed.
5.	Was the intervention(s) or treatment procedure(s) clearly described?	**Yes**	The orthodontic treatment approach and its main stages were clearly described.
6.	Was the post-intervention clinical condition clearly described?	**Yes**	The clinical outcome following orthodontic treatment was reported, allowing assessment of treatment effects.
7.	Were adverse events (harms) or unanticipated events identified and described?	**Yes**	Potential adverse outcomes related to root resorption during treatment were addressed by the authors.
8.	Was the follow-up information clearly reported?	**Unclear**	Follow-up was mentioned; however, the duration and structure of post-treatment observation were not clearly specified.

Article 2

Adélaïde C., de C. Elke V., Ria V., Nasser N.

Orthodontic–Surgical management in a class II case with idiopathic root resorption.

Journal of Oral Medicine and Oral Surgery.

DOI: 10.1016/j.jormas.2018.11.005

Table S2. JBI Critical Appraisal Checklist for Case Reports – Article 2

No.	JBI Critical Appraisal Question	Response	Explanation
1.	Were the patient's demographic characteristics clearly described?	**Yes**	The patient's age (14 years) and sex (female) were clearly reported.
2.	Was the patient's history clearly described and presented as a timeline?	**Yes**	The clinical history and the sequence of diagnostic, orthodontic, and surgical interventions were described in a logical and chronological manner.
3.	Was the current clinical condition of the patient on presentation clearly described?	**Yes**	The initial clinical condition was described in detail, including the Class II malocclusion and the extent of idiopathic root resorption.
4.	Were diagnostic tests or assessment methods and the results clearly described?	**Unclear**	CBCT imaging was performed and radiographic findings were presented; however, explicit diagnostic criteria for idiopathic root resorption and a structured exclusion of secondary causes were not clearly described.
5.	Was the intervention(s) or treatment procedure(s) clearly described?	**Yes**	The orthodontic–surgical treatment protocol was clearly described, including the sequence and rationale of the combined approach.
6.	Was the post-intervention clinical condition clearly described?	**Yes**	The post-treatment clinical condition was reported, allowing evaluation of orthodontic and surgical outcomes.
7.	Were adverse events (harms) or unanticipated events identified and described?	**Unclear**	Although the authors stated that post-treatment clinical examination was comparable to the pretreatment evaluation, adverse or unanticipated events were not explicitly discussed.
8.	Was the follow-up information clearly reported?	**Yes**	Follow-up duration was clearly specified (9 months), and treatment progress was evaluated using low-dose CBCT imaging.

Article 3
Samara E., Kelly E., Walker R., Borumandi F.
Multiple idiopathic cervical root resorption: Case report of an unusual presentation.
Special Care in Dentistry.
DOI: 10.1111/scd.12539

Table S3 JBI Critical Appraisal Checklist for Case Reports – Article 3

No.	JBI Critical Appraisal Question	Response	Explanation
1.	Were the patient's demographic characteristics clearly described?	**Yes**	The patient's demographic characteristics were clearly reported, including age (16 years) and sex (female).
2.	Was the patient's history clearly described and presented as a timeline?	**Yes**	The clinical history and circumstances leading to diagnosis were described in a logical and chronological manner.
3.	Was the current clinical condition of the patient on presentation clearly described?	**Yes**	The clinical presentation and extent of multiple idiopathic cervical root resorption were clearly described, supported by clinical and radiographic findings.
4.	Were diagnostic tests or assessment methods and the results clearly described?	**Unclear**	Relevant clinical and radiographic findings were presented, and familial occurrence was excluded; however, explicit diagnostic criteria for idiopathic cervical root resorption and a systematic exclusion of other potential secondary causes were not clearly defined.
5.	Was the intervention(s) or treatment procedure(s) clearly described?	**Unclear**	The therapeutic management, including orthodontic treatment, was described only briefly, with limited detail regarding the treatment protocol and its rationale.
6.	Was the post-intervention clinical condition clearly described?	**Unclear**	Although long-term observation was reported, the description of the post-intervention clinical condition was not fully consistent, particularly regarding the timing of treatment completion relative to follow-up assessments.
7.	Were adverse events (harms) or unanticipated events identified and described?	**Unclear**	The manuscript refers to potential future outcomes (e.g., possible need for extraction), but adverse events occurring during treatment or follow-up were not explicitly reported or discussed.

8.	Was the follow-up information clearly reported?	**Yes**	Follow-up information was clearly reported, including a recent panoramic radiograph and a stated follow-up duration of 4.5 years.

Article 4
Çelik Ö., Ersöz M., Kamalak H.
The Orthodontic Treatment of a Patient with Idiopathic Root Resorption in Upper Incisors.
Turkish Journal of Orthodontics.
DOI: 10.5152/TurkJOrthod.2016.15-00023R1

Table S4. JBI Critical Appraisal Checklist for Case Reports – Article 4

No.	JBI Critical Appraisal Question	Response	Explanation
1.	Were the patient's demographic characteristics clearly described?	**Yes**	The patient's age (14 years) and sex (female) were clearly reported.
2.	Was the patient's history clearly described and presented as a timeline?	**Yes**	The clinical history and course of orthodontic treatment were described in a logical chronological sequence.
3.	Was the current clinical condition of the patient on presentation clearly described?	**Yes**	The initial clinical condition, including idiopathic root resorption affecting the upper incisors, was clearly described.
4.	Were diagnostic tests or assessment methods and the results clearly described?	**No**	Diagnostic assessment methods and explicit criteria supporting the diagnosis of idiopathic root resorption were not clearly described, nor was a structured exclusion of secondary causes provided.
5.	Was the intervention(s) or treatment procedure(s) clearly described?	**Yes**	The orthodontic treatment approach and its main stages were clearly described.
6.	Was the post-intervention clinical condition clearly described?	**No**	Information regarding post-treatment clinical condition and outcomes was not reported.
7.	Were adverse events (harms) or unanticipated events identified and described?	**No**	Adverse or unanticipated events were not reported or discussed, and no explicit statement regarding harms was provided.
8.	Was the follow-up information clearly reported?	**No**	No follow-up period or post-treatment observational data were reported.
